# The effects of early short-term insulin treatment vs. glimepiride on beta cell function in newly diagnosed type 2 diabetes with HbA1c above 9%

**DOI:** 10.55730/1300-0144.5616

**Published:** 2023-01-20

**Authors:** Jelena STOJANOVIC, Marina ANDJELIC JELIC, Miljanka VUKSANOVIC, Milica MARJANOVIC PETKOVIC, Biljana JOJIC, Marko STOJANOVIC, Teodora BELJIC ZIVKOVIC

**Affiliations:** 1 Division of Endocrinology, Diabetes and Metabolic Disorders, Department of Internal Medicine, Zvezdara University Medical Center, Belgrade, Serbia; 2 Department of Internal Medicine, Faculty of Dental Medicine, University of Belgrade, Belgrade, Serbia; 3 Department of Internal Medicine, Medical Faculty, University of Belgrade, Belgrade, Serbia; 4 Department of Neuroendocrinology, Clinic for Endocrinology, Diabetes and Metabolic Diseases, University Clinical Center of Serbia, Belgrade, Serbia

**Keywords:** Early short-term insulin treatment, beta cell function, diabetes type 2, standardized test meal

## Abstract

**Background/aim:**

Type 2 diabetes mellitus (T2D) is a complex metabolic impairment. Beta cell (BC) failure is the most challenging among its pathogenetic mechanisms. Recognizing reversible contributors to BC failure could guide individualized approach to early T2D treatment. The aim of this study was to compare early short-term insulin treatment vs. glimepiride, both added to metformin, on BC function, glycemic and lipid control, during 12-month follow-up.

**Patients and methods:**

Eighty newly diagnosed T2D patients, 30–65 years of age, presenting with HbA1c ≥ 9% were enrolled in the study. They were randomly assigned to single-month initial insulin therapy (INS) added to metformin, or to glimepiride and metformin (OAD) as only treatment. Subjects assigned to initial insulin intervention were thereafter switched to OAD. C-peptide (C-Pep) was analyzed at baseline and 2 hours after standardized test meal (STM). All subjects were STM-retested after 3 and 12 months. HbA1c, serum lipids, BMI, HOMA IR, and HOMA B were assessed over follow-up.

**Results:**

HbA1c was lower in INS vs OAD at 3-months: 6.26 ± 0.18% vs 6.78 ± 0.10% (p = 0.016), remaining so by 12 months (p = 0.056). BMI-adjusted ΔC-Pep was greater in INS vs. OAD at 3 months (4.60 ± 0.59 vs. 3.21 ± 0.34 m^2^/kg; p = 0.044), persisting by 12 months (4.57 ± 0.56 vs. 3.04 ± 0.34 m^2^/kg; p = 0.023). Average ΔC-Pep improvement from recruitment to 3 months was 100.8% in INS, vs. 51.3% in OAD. Prevalence of STM-ΔC-Pep response greater than 2.4 ng/mL had risen 3.2-fold by 12 months in the INS, vs. 2.4-fold only in the OAD group (p = 0.018).

**Conclusion:**

Early short-term insulin intervention in newly diagnosed T2D improves beta cell function more than glimepiride, both added to metformin, resulting in a superior and longer lasting glycemic and lipid control.

## 1. Introduction

Global impact of type 2 diabetes mellitus (T2D) reaches pandemic proportions [1]. It is a complex metabolic impairment with multifactorial beta cell (BC) dysfunction early in its course [2]. Although T2D is a progressive disease, its remission might be possible, crucially depending on BC function [3–5]. BC function is compromised in part by potentially reversible factors, such as glucotoxicity and lipotoxicity [6,7]. Elevated glucose and lipid levels cause BC dysfunction and apoptosis through several mechanisms. Chronic inflammation is involved with dysregulation of numerous inflammatory cytokines (IL-1β, IL-6, IL-8, TNF-α, NF-κB, IFN-γ, CCL2, MCP1, and CXCL1) [8]. Endoplasmic reticulum is affected by oxidative stress damage [9]. Epigenetic alterations contribute with DNA methylation, histone modification, and alterations in noncoding RNAs. [10]. Targeting reversible contributors to BC failure could unlock possibilities for disease-modifying treatment in T2D [11,12].

Over the course of T2D progression, increasing doses and number of glucose-lowering medications are typically required, often leading to introduction of insulin treatment, once BC capacity is apparently exhausted. Such insulin treatment represents a form of exogenous replacement of a hormonal deficiency. On the other hand, early insulin treatment aimed at rapid elimination of glucotoxicity and BC recovery could represent a form of disease-modifying treatment [13,14]. Oral antidiabetics are also capable of swift glycemic improvement but do not match corresponding effects of insulin. These differences are attributed to additional antiinflammatory and antiapoptotic effects of insulin [14,15]. Insulin may also improve glucose-dependent insulinotropic polypeptide (GIP) reactivity to oral nutritional stimulation and alpha-cell recovery [16].

Affordable but reliable testing of BC secretory function is still elusive. Oral-based dynamic tests are attractive due to ease of clinical use and incorporation of incretin activation effects [17–21]. Oral food intake stimulates GIP and glucagon-like peptide-1 (GLP-1), leading to a 70% higher insulin response [22–24]. The most widely used food-based test of BC function, the mixed meal tolerance test, is hypercaloric and liquid-based, thus possibly diverging from the real-life solid-food impact on insulin activation [25–27]. Another approach is a fixed calories test hampered by bias related to patient height [27]. Standardized test meal (STM), as an in-house modification of the test introduced by Pozznan et al. in 1997, was investigated at our research center to assess residual insulin secretion in T2D patients, with the assessment of C-Peptide (C-Pep) for BC response [28].

The aim of our study was to assess beta cell (BC) secretory response to a standardized test meal in newly diagnosed T2D subjects and to compare the effect of early short-term insulin treatment vs. glimepiride, both added to metformin, on BC function, glycemic control and lipid metabolism, during a 12-month follow-up.

## 2. Patients and methods

This prospective, interventional randomized study, approved by Ethics Board of Zvezdara University Medical Center (Decision dated 29.01.2019), included 80 newly diagnosed T2D patients with initial HbA1c above 9.0%. They were recruited from the outpatient diabetes clinic of the Zvezdara University Medical Center in Belgrade, during the period of February 2019 to March 2020. Upon signing the informed consent form, they had 3 daily visits at the Center. On the first day, an STM was performed. Thereafter, patients were randomly assigned to receive either: metformin 2000 mg daily (or maximal tolerable dose) and glimepiride, or metformin with a 1-month course of daily basal or twice daily biphasic human insulin. Both patients and investigators were blinded for individual baseline C-peptide values. Randomization of patients was done on consecutive basis. The subjects were educated for insulin application by pens, proper diet, and physical activity. They were provided with meters for blood glucose (BG) self-monitoring, before each main meal, at bedtime and upon awakening. On the second and third days, after discussion of monitored BG levels, doses of insulin and glimepiride were adjusted. Subjects on insulin treatment were advised to continue monitoring BG levels and to report values below 4 mmol/L or above 10 mmol/L, for dose adjustment.

The exclusion criteria for the investigated patients encompassed: pregnancy or lactation, decreased renal function below eGFR of 60 mL/min/1.73 m^2^, liver impairment manifested by LFT elevation above 2 ULN, severe heart failure (NYHA III–IV), ketonuria exceeding trace amounts, systemic corticosteroid treatment in prior 3 months, any active or historical malignancy, other severe intercurrent acute illness, and autoimmune DM pathogenesis – indicated by elevated serum GAD or IA-2 antibodies. None of the 80 patients received hypolipemic treatment throughout the course of the study. Further study visits were performed after 1 month and 3, 6, 9, and 12 months. After 1 month, BG readings were evaluated. Insulin therapy was replaced by glimepiride. The subjects initially on metformin and glimepiride, continued with the same treatment, with glimepiride doses adjusted if necessary.

All patients were tested by a standardized test meal (STM) before treatment and at 3- and 12-month visits. STM consisted of a white-flour bread-roll (24 g of carbohydrates) and 200 mL 2.8% milk-fat yoghurt (12 g of carbohydrates). Upon an overnight fast, serum glucose and C-Pep were analyzed before and 2 hours after STM, which was consumed over 5 min [28]. At 3- and 12-month visits, oral antidiabetic treatment was omitted for 2 days prior to STM, to minimize confounding effect on analyzed parameters. Further assessment at baseline and at 3, 6, 9, and 12 months included body weight and height (with BMI calculation), biochemical serum analysis of HbA1c, total serum cholesterol (TC), HDL cholesterol (HDL-C), LDL cholesterol (LDL-C), and serum triglycerides (TG). Atherogenic index of plasma (AIP) was calculated using the formula AIP = log (TG/HDL-C). As a marker of plasma atherogenicity, AIP is considered predictive of atherosclerosis and of coronary heart disease risk [29]. According to their baseline BMI, patients were classified as normally nourished (NN) with BMI of 18.5–24.9 kg/m^2^, overweight (OW; BMI of 25.0–29.9 kg/m^2^) and obese (OB; BMI of > 30.0 kg/m^2^).

### 2.1. Calculations and statistical analysis

Serum C-Peptide (C-Pep) from all samples was analyzed by ECLIA assay (Cobas Elecsys C-Peptide assay, Roche Diagnostics GmbH, Mannheim, Germany) in Beo-Lab laboratory, Belgrade (baseline normal C-Pep reference range: 1.10–4.40 ng/mL). Absolute increase in serum C-Pep (ΔC-Pep) was calculated as difference between postprandial and baseline C-Pep (ng/mL). ΔC-Pep greater than 2.4 ng/mL was considered significant [25,28]. Relative C-Pep increase (ΔC-Pep%) was expressed as a ratio of ΔC-Pep to baseline C-Pep (in %). BMI-adjusted relative ΔC-Pep (BMDCP) was calculated for each patient as (ΔC-Pep%/BMI) × 100 (m^2^/kg).

Postprandial C-Pep to postprandial glucose ratio (PCPG) was analyzed as a marker of beta cell reserve (mcg/mmol) [30]. Change in PCPG over follow-up was analyzed as a ratio of PCPG at 12 months to PCPG at baseline, expressed in % (PCPG%). HOMA-IR and HOMA-B were calculated based on the following formulas modified for C-peptide, which included fasting plasma glucose (FPG in mmol/L) and fasting C-peptide (FCP in ng/mL) [31]:

HOMA-IR = 1.5 + (FPG × FCP × 331.1) / 2800; HOMA-B = 0.27 × FCP × 331.1 / (FPG - 3.5).

Results were presented as count (%), mean ± standard error of mean. Groups were compared for significance of difference using parametric (t-test) and nonparametric (chi-square, Mann–Whitney U, and Friedman) tests. To assess significance of correlation between variables, Pearson and Spearman correlations were used. SPSS Statistics v. 26 software was employed for the statistical analyses. p-values of less than 0.05 were regarded as indicating statistical significance.

## 3. Results

A total of 80 newly diagnosed T2D patients were included in the analysis, 58.8% male and 41.3% female. All subjects completed the 12-month follow-up period with no dropouts. Their average age at baseline was 54.04 ± 9.41 years, and the average BMI was 29.74 ± 4.91 kg/m^2^. Average baseline C-Pep was 2.29 ± 0.14 ng/mL, with average increase after the STM (ΔC-Pep) of 1.40 ± 0.20 ng/mL. ΔC-Pep of less than 2.4 ng/mL was observed in 85% of subjects at baseline, indicating impaired average beta cell reserve.

Baseline C-Pep did not differ between sexes (females vs males: 2.48 ± 0.26 ng/mL vs 2.16 ± 0.15 ng/mL, p = 0.246). Upon STM, a difference in response was noted between females and males. Absolute ΔC-Pep (1.92 ± 0.33 ng/mL vs 1.04 ± 0.14 ng/mL, p = 0.030) and relative ΔC-Pep% (0.80 ± 0.14% vs 0.50 ± 0.08%; p = 0.048) were both higher in females.

**Figure 1:**
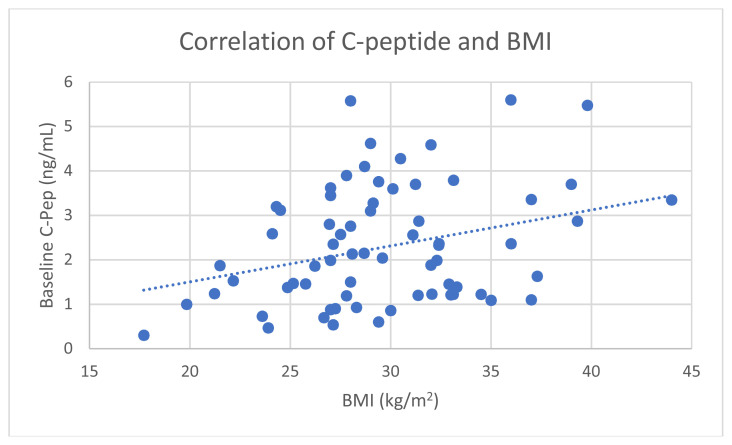
Correlation of baseline C-Pep with BMI (Pearson correlation coefficient r = 0.32; p = 0.004).

**Figure 2:**
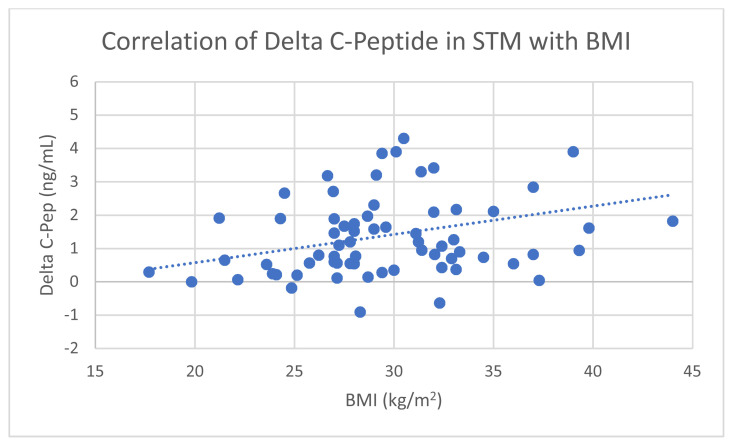
Correlation of Δ C-Pep in STM with BMI (Pearson correlation coefficient r = 0.23; p =0.043).

Average baseline C-Pep in all 80 newly diagnosed T2D patients positively correlated with BMI (Figure 1). Absolute increase in C-Pep (ΔC-Pep) during STM also positively correlated with BMI in all subjects (Figure 2). However, baseline C-Pep and ΔC-Pep did not correlate with baseline HbA1c or patient’s age.

Subjects that were normally nourished (NN), overweight (OW), or obese (OB) did not differ in age. Baseline C-Pep was lower in NN, compared to OW (p = 0.050) and OB (p = 0.020). There was no difference in baseline C-peptide levels between OW and OB subjects (Table 1). STM-stimulated increase of C-Pep (ΔC-Pep) at pretreatment visit was lower in NN (p = 0.046) compared to OB (Table 1). Baseline HbA1c was lower in the OB compared to NN (p = 0.019). Baseline HbA1c negatively correlated with baseline BMI overall in the investigated group of newly diagnosed T2D patients (Pearson correlation coefficient r = −0.34; p = 0.002). HOMA-IR did not differ in NN vs OW or OB. HOMA-B was greater in the OW (p = 0.007) and OB (p = 0.002) compared to NN. (Table 1)

### 3.1. Metabolic changes in newly diagnosed T2D patients on short-term insulin treatment or glimepiride, during the 12 months of follow-up

Forty-two patients were randomly assigned to receive early short-term insulin treatment added on metformin (INS). They did not differ in age, sex, nor BMI from 38 patients assigned to treatment with glimepiride and metformin (OAD). The two groups did not differ in baseline FPG, HbA1c, or serum lipid levels (Table 2).

**Figure 3:**
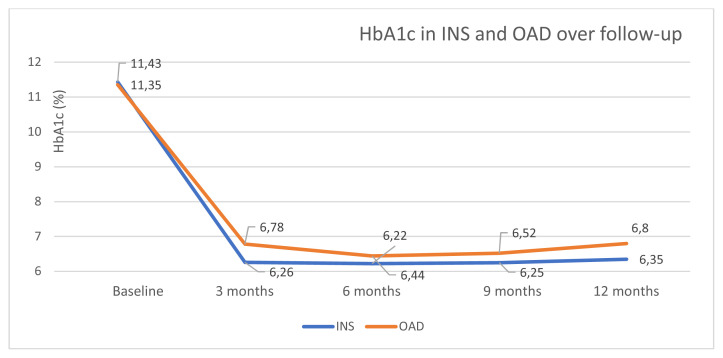
HbA1c changes on early insulin treatment (INS) vs. oral antidiabetics-only (OAD) groups during 12 months of follow-up.

In both INS and OAD groups, initial HbA1c improved after 3 months, remaining so by 12 months. Early single-month insulin treatment resulted in better glycemic control at 3 months compared to OAD: HbA1c 6.26 ± 0.18% vs. 6.78 ± 0.10% (p = 0.016). The difference in HbA1c was maintained in favor of the INS group during the 12-month follow-up (Figure 3).

There was no difference in average BMI between the two groups (28.36 ± 0.69 vs. 32.23 ± 2.07 kg/m^2^; p = 0.069) after 12 months. However, over the course of the study, the average BMI of INS group decreased by 3% (Table 3), while the average BMI of the OAD group increased by 5.7% (Table 4).

All investigated lipid markers, except HDL-cholesterol (HDL-C), improved after 3 months in both treatment groups, remaining so after 12 months in the INS group (Table 3). However, the initial improvement in LDL-cholesterol (LDL-C) at 3 months did not last for the follow-up of 12 months in the OAD group (Table 4). The changes in other serum lipids— triglycerides (TG), total cholesterol (TC), HDL-C, and atherogenic index of plasma (AIP)—were not different between the two groups over follow-up.

### 3.2. Beta cell function in newly diagnosed T2D on early short-term insulin treatment or on glimepiride added to metformin, over 12 months of follow-up

ΔC-Pep in INS group increased after 3 and 12 months, compared to the pretreatment values. The INS group improved STM-derived relative C-Pep (ΔC-Pep%) by 3 months (p = 0.000) and preserved this by 12 months (p = 0.000). The postprandial C-Pep to glucose ratio (PCPG) was also increased in the INS group at 3 months (p = 0.000) and remained so by 12 months (p = 0.000) (Table 5). A greater-than-two-fold improvement in average ΔC-Pep by 3 months was further increased to 7.4% by 12 months. Prevalence of subjects achieving ΔC-Pep greater than 2.4 ng/mL, following STM, increased after 12 months by 3.2-fold (11.9% to 38.1%) in the INS group.

In the OAD group, ΔC-Pep increased from its pretreatment value after 3 (p = 0.000) and 12 months (p = 0.001). The OAD group improved STM-derived relative C-Pep increase (ΔC-Pep%) at 3 months (p = 0.000), preserving this by 12 months (p = 0.000). The PCPG at 3 and 12 months in the OAD group was increased compared to pretreatment testing (p = 0.000) Table 6. Contrary to INS, the OAD group demonstrated a lower initial rise of ΔC-Pep from pretreatment to 3 months (51.3%), followed by a decrease in average ΔC-Pep (by 4.8%) in 12 months. Prevalence of subjects achieving ΔC-Pep greater than 2.4 ng/mL following STM, increased after 12 months by 2.4-fold (18.4% to 44.7%) in the OAD group (p = 0.018).

**Figure 4:**
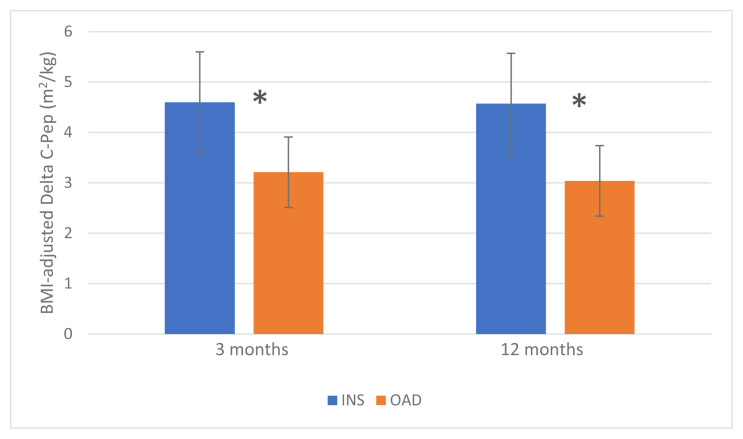
Differences in BMI-adjusted STM-stimulated relative C-peptide increase in short-term early insulin treatment vs. oral antidiabetics-only group after 3 and 12 months.

Our analysis of the pretreatment STM-derived C-Pep response in the whole group of newly diagnosed T2D patients revealed its strong correlation to BMI. Thus, we have further compared results from the two groups by introducing the BMI-adjusted STM-stimulated relative C-Pep increase (BMDCP). This marker was greater at 3 months in the INS compared to the OAD group (4.60 ± 0.59 vs. 3.21 ± 0.34 m^2^/kg; p = 0.044) and the difference persisted by 12 months (4.57 ± 0.56 vs. 3.04 ± 0.34 m^2^/kg; p = 0.023) (Figure 4).

There was no correlation of average AIP with any of the parameters of C-Pep response to STM: ΔC-Pep, ΔC-Pep%, PCPG—neither for the whole investigated cohort, nor for either sex separately.

## 4. Discussion

Results of our study support early 1-month insulin and metformin treatment’s superiority over glimepiride and metformin in newly diagnosed T2D, on beta cell (BC) functional recovery, glycemic and lipid control, enduring beyond the short span of treatment itself. The concept of early short-term insulin treatment in newly diagnosed T2D shifts away from the classic role of insulin at a later-stage of T2D. This early intervention may promote BC functional recovery, pointing to a disease-modifying effect of insulin treatment [11–14].

Our results support previously reported advantages of early short-term insulin compared to oral-only treatment regarding glycemic and lipid control, extending beyond the brief span of treatment itself [5,32,33].

Early short-term insulin treatment in newly diagnosed T2D may reverse gluco- and lipotoxicity, as main indirect contributors of BC dysfunction [11]. It decreases insulin resistance [12]. In addition to its glucose lowering effects, insulin has direct antiinflammatory and antiapoptotic effects on BC [12,15]. It also reduces postchallenge hyperglucagonemia [16] and improves endogenous insulin secretion [11,12]. Reversal in BC dedifferentiation was observed after insulin treatment [12]. Accumulating research evidence announces possible repositioning of insulin therapy as disease-modifying treatment [11]. Despite all these reported benefits, reluctance is often prevalent regarding initiating insulin treatment in T2D. Obstacles may include limited access to insulin or monitoring tools, necessity for additional caregiver and patient training, or the fear of hypoglycemia [12].

C-Pep response augmentation parallel to glycemic control improvement, as observed in both of our treatment groups, highlights the universal importance of rapid elimination of glucotoxicity as instrumental in beta cell (BC) function recovery [14, 34–36]. The observed greater benefits of early short-term insulin were demonstrated by continually improving C-Pep response over follow-up in addition to superior glycemic and lipid control, long outlasting the course of insulin treatment itself, with neutral BMI impact. On the contrary, the decrease in C-Pep response in the OAD group from 3 to 12 months could be attributed to secretagogue effect on BC exhaustion.

Unlike comparable previous studies designed with either intensive insulin treatment with multiple daily injections (MDI) or continuous subcutaneous insulin infusion (CSII) [6,14,16], we have used once basal or twice daily biphasic human insulin, for the initial single-month intervention. We hoped to provide easier insulin application and titration, with avoidance of hypoglycemia. Ryan et al. found the impact of early insulin treatment with MDI (2 or 3 weeks) on body weight as neutral, compared to oral treatment upon 1-year follow-up in the investigated group of 16 newly diagnosed T2D patients [13]. This study involved a smaller number of subjects with higher average BMI and shorter duration of insulin treatment than in our study. Alvarsson et al. demonstrated improvement of glucagon-stimulated C-Pep response after 1, 2, 4, and 6 years of insulin intervention compared to orally treated recently diagnosed T2D patients, concluding that insulin treatment alleviates beta cell (BC) secretory demands [34,35]. Their cohort was smaller than ours, with higher male prevalence, and smaller average BMI, and it included patients nonnaïve to treatment with average initial HbA1c of 6.8% or 7.1% (contrary to our newly diagnosed, treatment-naïve patients with HbA1c > 9.0%). Their follow-up period was longer than in our study, but glucagon test was used to assess BC function, whereas we used standardized test meal as more convenient. Early intensive insulin treatment (CSII or MDI) proved superior to oral-only in meeting and maintaining the target of prolonged T2D remission upon discontinuation of 2–3-week-long initial treatment in the pivotal multicentric study by Weng J et al. [14]. One-third of the subjects in this 382-patient strong cohort were assigned to CSII, which is not readily available in our practice. HOMA-B in IVTT was used by Weng et al. to assess BC function, which we found impractical. Their average patient BMI was in high-normal range, compared to prevalent obesity in our cohort. Despite several study design differences, comparative strengths and weaknesses, and geographical background specificities, our results do support and extend these previous reported findings, reaffirming them in a real-life context of more practical investigation and more convenient selection of insulin treatment modalities.

Due to limited duration and lower insulin dose requirements, the concept of early short-term insulin intervention leads to a smaller hypoglycemic risk and less weight gain than the classical late-stage, long-term insulin treatment, as is observed in our study. Furthermore, our preference for basal-insulin-only simplifies the insulin intervention in newly diagnosed T2D with high HbA1c. Our results should encourage practical implementation of early short-term insulin intervention in T2D.

To the best of our knowledge, none of the diabetes treatment guidelines incorporate BC function assessment as input for therapeutic algorithms. However, heterogeneity is well-recognized among newly diagnosed T2D patients regarding causal predominance of insulin resistance or BC failure [37]. Dynamic C-Pep tests are reported as prognostic of treatment response [38]. Results provided by a simple and practical tool, such as STM, offer valuable insight into BC secretory reserve and may shape individual approach to treatment. STM is an affordable and reliable tool for BC investigation in practical outpatient setting, with factoring in its dependency on sex and BMI. As a solid-food-based test, STM should superiorly approximate real-life stimulation by incorporating incretin activation, and deserves full consideration as a simple alternative for in vivo BC assessment [20–28].

We report of significantly greater C-Pep response to STM in females. Previous data from the mixed meal tolerance test indicated absence of sex-based difference in C-Pep response [39]. However, some explanatory basis for our observation could be found in sex C-Pep dimorphism observed in newborns. Higher serum C-Pep in girls was attributed to inherently higher insulin resistance [40]. Previously reported higher serum glucagon (both fasting and postprandial) and postprandial serum leptin in males, could also support our observations [41].

The association of BMI and postprandial hormonal response is complex and most probably multidimensional. Higher baseline and postprandial insulin but with a delayed peak were observed in the obese [41]. Obesity could result in increased insulin secretion pursuing to neutralize rising insulin resistance. Postmortem studies in both diabetics and nondiabetics reported increased beta cell (BC) mass in the obese [42]. Furthermore, increased body fat was recently also associated with better preserved BC secretory response, attributed to gluco-lipotoxicity-induced adaptation [43]. In the large multicentric study by Weng J et al., newly diagnosed T2D patients, achieving remission after short intensive insulin treatment, had significantly higher average initial BMI than those failing to achieve remission [14]. The average pretreatment HbA1c of our patients, inversely correlated with their BMI. Normally nourished subjects did not differ from the overweight and obese in the average HOMA-IR, but demonstrated a lower HOMA-B and lower relative pretreatment C-peptide response to STM.

In prior studies, atherogenic index of plasma (AIP) was credited as a marker of BC dysfunction, inversely correlating with C-Pep secretion, and possibly predicting the need for insulin treatment [44,45]. We found no correlation of AIP to parameters of baseline or STM-stimulated C-Pep either at the initial testing or during follow-up. Possible limitations to conclusions of our study include the uncertainty about true duration of individual glucose impairment predating T2D diagnosis, as glucotoxicity could have attenuated the postprandial C-Pep rise. Study subjects were not homogenized for glimepiride or insulin doses nor for initial HbA1c levels, which could also contribute to potential limitations of the study. Glimepiride could also have impacted objective beta cell function assessment by affecting insulin and C-Pep secretion. Other antidiabetic drugs (including SGLT-2 inhibitors, DPP4 inhibitors, or GLP-1 receptor agonists) were not used in the study, since they were unavailable through prescription reimbursement.

## 5. Conclusion

Early short-term insulin treatment in newly diagnosed treatment-naïve T2D patients improves beta cell function more than long-term therapy with glimepiride, as reflected in C-peptide response to standardized test meal. Limited insulin intervention added to metformin is superior to glimepiride and metformin treatment regarding glycemic and lipid control. Legacy of its benefits extends to after the intervention duration, without a negative impact on body weight. Convenient insight into beta cell capacity at diagnosis and its response to treatment through a standardized test meal may guide individual approach amidst the known heterogeneity of T2D patients.

## Figures and Tables

**Table 1:** Age, HbA1c, Baseline C-Pep, ^Δ^C-Pep, HOMA-IR, and HOMA-B differences between different BMI categories of newly diagnosed T2D subjects.

	Normally nourished, NN *(n = 12)* BMI: 18.5–24.9 kg/m^2^	Overweight, OW *(n = 32)* BMI: 25.0–29.9 kg/m^2^	Obese, OB *(n = 36)* BMI: >30.0 kg/m^2^	NN vs OW	NN vs OB
*Age (years)*	54.58 ± 2.97	55.03 ± 1.61	52.97 ± 1.59	*p = 0.896*	*p = 0.638*
*Baseline HbA1c (%)*	**12.42 ± 0.50**	**11.48 ± 0.22**	**10.97 ± 0.27**	***p = 0.051***	***p = 0.019***
Baseline C-Pep (ng/mL)	**1.61 ± 0.28**	**2.33 ± 0.22**	**2.48 ± 0.21**	***p = 0.050***	***p = 0.020***
**Δ**C-Pep (ng/mL)	**0.74 ± 0.26**	1.26 ± 0.18	**1.74 ± 0.41**	*p = 0.117*	***p = 0.046***
*HOMA-IR*	4.26 ± 0.48	5.14 ± 0.42	5.14 ± 0.32	*p = 0.178*	*p = 0.142*
*HOMA-B*	**13.51 ± 2.62**	**24.34 ± 2.67**	**31.79 ± 4.81**	***p = 0.007***	***p = 0.002***

ΔC-Pep, absolute increase in C-peptide; HOMA-IR, homeostatic model assessment of insulin resistance;

HOMA-B, homeostasis model assessment of β-cell function.

**Table 2:** Baseline demographic and metabolic characteristics in INS and OAD groups.

	INS group	OAD group	p
Age (years)	54.12 ± 1.62	53.94 ± 1.33	*0.936*
Sex (Males %)	64.3%	52.6%	*0.655*
BMI (kg/m^2^)	29.2 ± 0.74	30.34 ± 0.81	*0.305*
FPG (mmol/L)	13.61 ± 0.60	13.14 ± 0.53	*0.558*
HbA1c (%)	11.43 ± 0.19	11.34 ± 0.15	*0.816*
TC (mmol/L)	6.25 ± 0.26	5.94 ± 0.20	*0.356*
TG (mmol/L)	3.93 ± 0.69	3.24 ± 0.29	*0.378*

FPG, fasting plasma glucose; TC, total cholesterol; TG, triglycerides.

**Table 3:** BMI and serum lipids during study follow-up in the INS group

	Baseline	3 months	6 months	9 months	12 months	Baseline vs. 3 months	Baseline vs. 12 months
BMI (kg/m^2^)	**29.20 ± 0.74**	**28.34 ± 0.66**	28.34 ± 0.66	28.46 ± 0.66	**28.36 ± 0.69**	***p = 0.018***	***p = 0.043***
TC (mmol/L)	**6.25 ± 0.26**	**5.10 ± 0.17**	4.97 ± 0.18	4.93 ± 0.16	**4.82 ± 0.14**	***p = 0.000***	***p = 0.000***
LDL-C (mmol/L)	**3.30 ± 0.13**	**3.06 ± 0.13**	3.08 ± 0.16	3.03 ± 0.14	**2.99 ± 0.14**	***p = 0.000***	***p = 0.001***
HDL-C (mmol/L)	1.02 ± 0.05	1.00 ± 0.04	0.99 ± 0.04	0.98 ± 0.04	0.97 ± 0.04	*p =0.595*	*p = 0.357*
TG (mmol/L)	**3.93 ± 0.69**	**2.10 ± 0.14**	2.01 ± 0.22	1.91 ± 0.13	**2.01 ± 0.18**	***p = 0.011***	***p = 0.009***
AIP	**0.46 ** **± 0** **.06**	**0.31 ** **± 0.** **03**	0.26 ± 0.04	0.28 ± 0.03	**0.29 ** **± 0** **.04**	***p = 0.003***	***p = 0.017***

TC, total cholesterol; LDL-C, LDL-cholesterol; HDL-C, HDL-cholesterol; TG, triglycerides; AIP, atherogenic index of plasma.

**Table 4:** BMI and serum lipids during study follow-up in the OAD group.

	Baseline	3 months	6 months	9 months	12 months	Baseline vs 3 months	Baseline vs. 12 months
BMI (kg/m^2^)	**30.34 ± 0.81**	**26.49 ± 0.74**	29.51 ± 0.74	29.41 ± 0.75	32.23 ± 2.07	***p = 0.002***	*p = 0.324*
TC (mmol/L)	**5.94 ± 0.20**	**5.09 ± 0.11**	5.02 ± 0.10	4.84 ± 0.09	**4.90 ± 0.09**	***p = 0.000***	***p = 0.000***
LDL-C (mmol/L)	**3.15 ± 0.15**	**3.00 ± 0.11**	2.97 ± 0.10	2.89 ± 0.09	3.07 ± 0.23	***p = 0.038***	*p = 0.728*
HDL-C (mmol/L)	1.02 ± 0.04	1.02 ± 0.04	1.02 ± 0.04	1.02 ± 0.04	1.01 ± 0.04	*p = 0.761*	*p = 0.773*
TG (mmol/L)	**3.24 ± 0.30**	**1.98 ± 0.06**	2.06 ± 0.12	1.87 ± 0.05	**1.81 ± 0.06**	***p = 0.000***	***p = 0.000***
AIP	**0.46 ** **± 0** **.03**	**0.29 ** **± 0** **.02**	0.30 ± 0.03	0.27 ± 0.02	**0.26 ** **± 0** **.02**	***p = 0.000***	***p = 0.000***

TC, total cholesterol; LDL-C, LDL-cholesterol; HDL-C, HDL-cholesterol; TG, triglycerides; AIP, atherogenic index of plasma.

**Table 5:** Beta cell function during study follow-up in the INS group.

	Baseline	3 months	12 months	Baseline vs. 3 months	Baseline vs. 12 months
Preprandial C-peptide (ng/mL)	1.59 ± 0.14	1.71 ± 0.15	1.94 ± 0.18	*p = 0.382*	*p = 0.059*
Postprandial C-peptide (ng/mL)	**2.54 ± 0.27**	**3.72 ± 0.34**	**4.10 ± 0.34**	***p = 0.000***	***p = 0.000***
^Δ^C-Pep (ng/mL)	**0.96 ± 0.17**	**2.01 ± 0.24**	**2.16 ± 0.22**	***p = 0.000***	***p = 0.000***
^Δ^C-Pep% (%)	**63.71 ± 12.87**	**127.28 ± 15.52**	**124.86 ± 14.80**	***p = 0.000***	***p = 0.000***
Preprandial glycemia (mmol/L)	**13.61 ± 0.60**	**7.11 ± 0.23**	**6.96 ± 0.29**	***p = 0.000***	***p = 0.000***
Postprandal glycemia (mmol/L)	**17.75 ± 0.69**	**9.93 ± 0.45**	**9.66 ± 0.41**	***p = 0.000***	***p = 0.000***
PCPG (mcg/mol)	**0.17 ± 0.02**	**0.41 ± 0.04**	**0.46 ± 0.04**	***p = 0.000***	***p = 0.000***
HbA1c (%)	**11.43 ± 0.19**	**6.26 ± 0.18**	**6.35 ± 0.15**	***p = 0.000***	***p = 0.000***
BMDCP (m^2^/kg)	**4.44 ± 0.57**	**4.60 ± 0.5**9	4.57 ± 0.56	***p = 0.050***	*p = 0.681*

ΔC-Pep, absolute increase in C-peptide; ΔC-Pep%, relative increase in C-peptide;

PCPG, postprandial C-Pep to postprandial glucose ratio; BMDCP- BMI-adjusted relative ΔC-Pep.

**Table 6:** Beta cell function during study follow-up in the OAD group.

	Baseline	3 months	12 months	Baseline vs. 3 months	Baseline vs. 12 months
Preprandial C-peptide (ng/mL)	3.07 ± 0.18	3.15 ± 0.16	2.95 ± 0.14	*p = 0.405*	*p = 0.275*
Postprandial C-peptide (ng/mL)	**4.96 ± 0.48**	**6.01 ± 0.44**	**5.68 ± 0.38**	***p = 0.001***	***p = 0.029***
^Δ^C-Pep (ng/mL)	**1.89 ± 0.38**	**2.86 ± 0.37**	**2.73 ± 0.33**	***p = 0.000***	***p = 0.001***
^Δ^C-Pep% (%)	**61.41 ± 8.10**	**94.18 ± 10.29**	**95.66 ± 11.20**	***p = 0.000***	***p = 0.000***
Preprandial glycemia (mmol/L)	**13.14 ± 0.53**	**7.93 ± 0.33**	**7.29 ± 0.30**	***p = 0.000***	***p = 0.000***
Postprandal glycemia (mmol/L)	**15.52 ± 0.77**	**10.58 ± 0.37**	**9.88 ± 0.38**	***p = 0.000***	***p = 0.000***
PCPG (mcg/mol)	**0.39 ± 0.06**	**0.61 ± 0.06**	**0.63 ± 0.06**	***p = 0.000***	***p = 0.000***
HbA1c (%)	**11.35 ± 0.30**	**6.78 ± 0.10**	**6.80 ± 0.18**	***p = 0.000***	***p = 0.000***
BMDCP (m^2^/kg)	**3.09 ± 0.31**	**3.21 ± 0.34**	3.04 ± 0.34	***p = 0.015***	*p = 0.840*

ΔC-Pep, absolute increase in C-peptide; ΔC-Pep%, relative increase in C-peptide;

PCPG, postprandial C-Pep to postprandial glucose ratio; BMDCP- BMI-adjusted relative ΔC-Pep
